# Heterologous Expression and Rational Design of l-asparaginase from *Rhizomucor miehei* to Improve Thermostability

**DOI:** 10.3390/biology10121346

**Published:** 2021-12-17

**Authors:** Xian Zhang, Zhi Wang, Yimai Wang, Xu Li, Manchi Zhu, Hengwei Zhang, Meijuan Xu, Taowei Yang, Zhiming Rao

**Affiliations:** 1 The Key Laboratory of Industrial Biotechnology of Ministry of Education, School of Biotechnology, Jiangnan University, Wuxi 214122, China; zx@jiangnan.edu.cn (X.Z.); mfq1328248220@163.com (Z.W.); 1024180205@stu.jiangnan.edu.cn (Y.W.); 6180201061@stu.jiangnan.edu.cn (M.Z.); 6190205064@stu.jiangnan.edu.cn (H.Z.); xumeijuan@jiangnan.edu.cn (M.X.); yangtw@jiangnan.edu.cn (T.Y.); 2 School of Food Science and Technology, Shihezi University, Shihezi 832003, China; leeluok@163.com

**Keywords:** l-asparaginase, *Rhizomucor miehei*, heterologous expression, thermostability, molecular modification, rational design

## Abstract

**Simple Summary:**

l-asparaginase has been extensively applied in food industries. However, the application of l-asparaginase from non-thermophilic sources is greatly limited due to the poor thermostability and the complex environments typically encountered in food industries. Therefore, improving the thermostability of l-asparaginase is essential for its industrial application. In this work, the thermostability and enzyme activity of heterologously expressed l-asparaginase from *Rhizomucor miehei* was greatly improved by rational design and molecular modification. Moreover, we further characterized the mechanism underlying improved thermostability in detail. A high-yield l-asparaginase *B. subtilis* recombinant was constructed by 5′ untranslated region (UTR) modification. These results demonstrate that rational design can be an efficient approach for enhancing the thermostability of l-asparaginase from non-thermophilic sources.

**Abstract:**

l-asparaginase (EC 3.5.1.1) hydrolyzes l-asparagine to produce l-aspartate and ammonia and is widely found in microorganisms, plants, and some rodent sera. l-asparaginase used for industrial production should have good thermostability. We heterologously expressed l-asparaginase from *Rhizomucor miehei*, selected nine loci for site-directed mutagenesis by rational design, and obtained two mutants with significantly improved thermostability. The optimal temperature of mutants S302I and S302M was 50 °C. After incubating the mutant and wild-type enzymes at 45 °C for 35 h, the residual activity of the wild-type enzyme (WT) was only about 10%. In contrast, the residual activity of S302I and S302M was more than 50%. After combination mutagenesis, *Bacillus subtilis* 168-pMA5-A344E/S302I was constructed using the food-safe host strain *B. subtilis* 168. Additionally, a 5′ untranslated region (UTR) modification strategy was adopted to enhance the expression level of *R. miehei*-derived l-asparaginase in *B. subtilis*. In a 5-L fermenter scale-up experiment, the enzyme activity of recombinant *B. subtilis* 168-pMA5-UTR-A344E/S302I reached 521.9 U·mL^−1^ by fed-batch fermentation.

## 1. Introduction

l-asparaginase (EC 3.5.1.1) is an amide hydrolase that catalyzes the hydrolysis of l-asparagine to l-aspartic acid and ammonia [1]. This enzyme can be used to treat childhood acute leukemia and in the production of low-acrylamide foods [2,3]. In 1963, Broome [4] discovered l-asparaginase in guinea pig serum and attributed the antitumor properties of the latter reported by Kidd in 1953 [3] to this l-asparaginase activity. In 2002, researchers found that acrylamide, a carcinogenic substance, is generated during food processing at elevated temperatures. l-asparaginase has been investigated for its ability to catalyze the breakdown of l-asparagine, a precursor to acrylamide formation, without adverse effects on food flavor [5]. Several researchers have reported that the pretreatment of foods with l-asparaginase prior to high-temperature frying and baking reduced the levels of acrylamide in the final products [6,7].

l-asparaginase is widely distributed in nature and has been identified in plants, animals, and microorganisms [8]. Microorganisms, including bacteria, yeasts, and filamentous fungi, are ideal sources of l-asparaginase for food production and other applications because they are relatively inexpensive to culture and enable convenient optimization of the production and purification process. Moreover, enzymes isolated from these sources are relatively more stable than those extracted from plants or animals [9]. Mashburn and Wriston first obtained l-asparaginase from *Escherichia coli* in 1964 [10]. Since then, an increasing number of microorganisms have been identified for the production of l-asparaginase.

l-asparaginase preparations obtained from *E. coli* or *Erwinia chrysanthemi* have been extensively applied in the food and healthcare industries. In the food industry, l-asparaginase is mainly used in food pretreatment processes to remove l-asparagine, the precursors of acrylamide [6]. However, the pretreatment conditions show significant differences. Therefore, these applications have been hindered by the complex environments typically encountered in the food and healthcare industries, owing to the poor thermostability and limited substrate specificity of conventional l-asparaginase preparations. Numerous researchers have attempted to use rational design, directed evolution, and other molecular approaches to improve these properties, based on the reported catalytic mechanism and crystal structure.

In the healthcare industry, l-asparaginase is used to treat various diseases, such as Hodgkin’s lymphoma [11], acute lymphoblastic leukemia [12], and other malignancies of the lymphatic system [13]. In the food industry, l-asparaginase can reduce the production of the carcinogen acrylamide in fried or baked goods [6,7]. However, l-asparaginase that are already in the process of application are of increasing concern. In both the healthcare and food industries, the complexity of the environment requires enzymes to maintain high catalytic activity over wide ranges of pH and temperature. Several protein engineering approaches are available to overcome the limitations of natural enzymes and improve their industrial performance. In the molecular modification of l-asparaginase, rational design is the primary strategy for improving the enzymatic performance. Li et al. [14] reported that the mutation of *E. coli* l-asparaginase from aspartate to proline at position 178 improved the thermostability of the enzyme without affecting its catalytic activity. Furthermore, Kotzia and Labrou [15] constructed a gene mutant library for the l-asparaginase from *E. chrysanthemi* and *Erwinia carotovora*. Screening revealed that the D133V mutant displayed a half-life of 159.7 h at 50 °C, compared to 2.7 h at 50 °C for the wild-type enzyme (WT). Vidya et al. [16] investigated the influence of surface charge on the thermostability of *Escherichia* sp. l-asparaginase and found that fixed point mutation of the two positively charged residues K139 and K207 to neutral alanine residues (K139A and K207A) led to improved thermostability compared to the wild-type enzyme.

With the development of genetic engineering technology in recent years, recombinant DNA techniques have been adopted by an increasing number of researchers [17]. Researchers frequently use model systems with transparent genetic backgrounds, such as *E. coli*, *B. subtilis*, and *Pichia pastoris*, as host microorganisms to achieve the fermentation production of l-asparaginase. For example, Meena et al. [18] heterologously expressed the l-asparaginase gene *ansA* from *Streptomyces griseus* NIOT-VKMA29 in *E. coli* and reported that the enzyme activity yield increased by threefold to 123 U·mL^−1^ compared to fermentation of the original strain. Chityala et al. [19] ligated the glutaminase-free l-asparaginase gene from *Pectobacterium carotovorum* MTCC 1428 into the pHT43 plasmid and transformed it into *B. subtilis* WB600N, whereupon the l-asparaginase production reached 105 U·mL^−1^ after IPTG induction. Feng [20] cloned the l-asparaginase gene from *B. subtilis* using the shuttle plasmid pMA5 as the vector and *B. subtilis* WB600 as the host bacterium and reported that the enzyme displayed low glutaminase activity after expression and purification, while the enzyme yield reached 407.6 U·mL^−1^ in a 3-L fermenter. Cuong et al. [21] overexpressed the l-asparaginase gene from *E. chrysanthemi* NCPBB1125 in *P. pastoris* SMD1168 and *P. pastoris* X33. SDS-PAGE analysis confirmed the successful expression of the recombinant l-asparaginase, and the enzyme activity was measured to be 6.251 U·mg^−1^ following purification. Ferrara et al. [22] successfully expressed the l-asparaginase gene derived from *Saccharomyces cerevisiae* in *P. pastoris* and reported an eightfold increase in enzyme activity compared to fermentation of the original strain.

In previous research by our group, we heterologously expressed l-asparaginase from *Pyrococcus yayanosii* CH1 and *B. subtilis* with *E. coli* as the host bacterium, assayed its enzymatic properties, and modified it using rational design principles. However, the specific enzyme activity of l-asparaginase from *B. subtilis* was only 92.45 U·mg^−1^ [23], while the optimal temperature of l-asparaginase from *P. yayanosii* CH1 was 95 °C, neither of which is suitable for industrial applications. Therefore, screening and mining a more suitable l-asparaginase are essential for industrial and food applications. We previously reported an l-asparaginase mutant with significantly increased activity by site-directed mutation [24]. In the current work, to obtain a stable l-asparaginase by rational design in mutant libraries, computational design software (FoldX5) combined with conservativeness analysis and functional region evaluation was applied to predict potential stable point mutations. Then, the best variant was intracellularly overexpressed in *B. subtilis* 168. Molecular dynamics simulations were used to elucidate the mechanism underlying the stability enhancement. Finally, we obtained a combination mutant with high enzyme activity and good stability.

## 2. Materials and Methods

### 2.1. Plasmids, Strains, and Media

The strains and plasmids used in this study are summarized in Supplementary Table S1, and the primers used are listed in Supplementary Table S2. *Escherichia coli* BL21(DE3) and *B. subtilis* 168 were used as gene expression hosts, while *E. coli* JM109 was used for gene cloning. The methods for the transformation of the gene to *B. subtilis* is referred to by You et al. [25]. *Escherichia* *coli* cells were cultured at 37 °C in LB medium (10 g/L NaCl, 10 g/L peptone, and 5 g/L yeast extract), and *B. subtilis* 168 cells were grown at 37 °C in fermentation medium (47 g/L glycerin, 35 g/L yeast extract, 1.5 g/L NH_4_Cl, 5 g/L NaCl, 15 g/L corn steep liquor, 2.04 g/L KH_2_PO_4_, 2.61 g/L K_2_HPO_4_, 1.85 g/L MgSO_4_·7H_2_O, and 1 g/L l-asparagine). Kanamycin (50 μg/mL) was added when necessary. We used pET-28a and pMA5 for gene expression, which were kept in our laboratory. NdeI, XhoI, DpnI, Ex Taq DNA polymerase, and T4 DNA ligase were purchased from Takara (Dalian, China). Gel recovery kits and plasmid extraction kits were purchased from Jierui Bioengineering (Shanghai, China). Kanamycin, Nessler’s reagent, Tris base and isopropyl *β*-d-thiogalactopyranoside (IPTG) were purchased from Yuanye Bio-Technology (Shanghai, China). Glycerin, imidazole, sodium chloride, and l-asparagine were obtained from commercial suppliers and used as received.

### 2.2. Expression of l-asparaginase Genes from Various Sources in E. coli

After searching through NCBI (https://www.ncbi.nlm.nih.gov/, accessed on 23 November 2021), we selected the l-asparaginase genes from *Aspergillus flavus* (RMZ45850), *Cyberlindnera jadinii* (CEP24033), and *R. miehei* (AHF50151) (*Afans*, *Cjans*, and *Rmans*) for use in this work. We synthesized these sequences containing NdeI and XhoI restriction sites to obtain the target gene, and 6×Histag was added to the C-terminal of l-asparaginase for the protein purification. The gene fragments were obtained by PCR amplification. The plasmid pET-28a, after digestion with NdeI and XhoI, was ligated to the gene fragments by homologous recombination. The ligated products were transformed into the host *E. coli* BL21 cells, which were plated onto kanamycin-resistant plates and incubated until single colonies had formed. The pET-28a plasmid was verified using the universal primer of pET-28a, and positive transformants were picked and sent to GENEWIZ Biotechnology (Suzhou, China) for sequencing.

### 2.3. SDS-PAGE Analysis of Recombinant l-asparaginase

Each recombinant *E. coli* strain was inoculated into 10 mL of LB liquid medium containing 50 μg/mL kanamycin and cultured at 37 °C and 200 rpm for 12 h. Then, 500 μL of this starter culture was transferred to 50 mL of LB medium containing 50 μg/mL of kanamycin. The resulting culture was incubated at 37°C until the optical density at 600 nm (OD_600_) had reached 0.6–0.8, at which point IPTG was added to a final concentration of 0.5 mmol·L^−1^ and the induced culture was incubated at 25 °C for an additional 20 h. The cells were centrifuged at 8000× *g* and 4 °C for 5 min and resuspended in 10 mL of 50 mmol·L^−1^ phosphate buffer (pH 7.4) after washing three times. The resuspended cells were disrupted by sonication for 30 min, and the cell debris was discarded after centrifugation at 8000× *g* and 4 °C for 30 min. Finally, the expression of the target enzyme in the supernatant was analyzed by SDS-PAGE.

### 2.4. Purification and Quantification of Recombinant l-asparaginase

Each crude enzyme solution containing recombinant l-asparaginase with a His6 tag was purified by Ni-NTA affinity chromatography. The soluble supernatant fraction was first filtered and then loaded onto a 1 mL Ni-NTA affinity column that had been pre-equilibrated with 50 mmol·L^−1^ washing buffer (20 mmol·L^−1^ Tris and 500 mmol·L^−1^ NaCl, pH 7.4), and then elution buffer (20 mmol·L^−1^ Tris, 500 mmol·L^−1^ NaCl, and 500 mmol·L^−1^ imidazole, pH 7.4) was used to elute the unbound proteins and target protein with linear gradient elution. The gradient of the linear elution was set on the protein purified system, which increased the concentration of the eluate from 1% per milliliter to 100%, and we used Äkta PrimePlus to run the column. Finally, the target proteins were analyzed by SDS-PAGE and stored in 10% glycerol at −40 °C. The protein concentration was determined using the Bradford assay.

### 2.5. Determination of l-asparaginase Activity

The l-asparaginase activity was determined by measuring the amount of ammonia generated by the enzymatic reaction. One unit of l-asparaginase activity was defined as the amount of enzyme that produced 1 μmol of ammonia per minute. Specifically, 800 μL of Tris-HCl buffer (pH 7.0) containing a final concentration of 25 mmol·L^−1^ of the substrate l-asparagine was preheated in a water bath at 45 °C for 5 min. Next, 100 μL of the enzyme solution (to reach the enzyme activity of 0.05 mg·mL^−1^) was added. After incubation for a further 10 min, 100 μL of 15% trichloroacetic acid solution was added to terminate the reaction. The resulting solution was then centrifuged at room temperature for 10 min, and 200 μL of the supernatant was mixed with 200 μL of Nessler’s reagent and 4.8 mL of distilled water. After allowing the reaction to stand at room temperature for 10 min, the ammonia concentration was determined by measuring the absorbance at 450 nm using a UV spectrophotometer.

### 2.6. Simulation and Analysis of the Three-Dimensional Structure of l-asparaginase

To predict the three-dimensional structure of l-asparaginase, we submitted the amino acid sequence to SWISS-MODEL (http://swissmodel.expasy.org/, accessed on 23 November 2021). We used DNAMAN to compare the amino acid sequences of l-asparaginase from different sources, PYMOL to analyze *R. miehei* l-asparaginase, and GROMACS (version 5.0.2, http://www.gromacs.org/, accessed on 23 November 2021) to perform molecular dynamics simulations of the protein.

### 2.7. Site-Directed Mutagenesis

Targeted mutation of the *Rmans* gene was performed using reverse PCR with the constructed wild-type recombinant plasmid as the template, and mutation sites were introduced into the upstream and downstream primers. The PCR procedure consisted of the following steps: (1) pre-denaturation at 95 °C for 5 min, (2) denaturation at 95 °C for 45 s, (3) annealing for 30 s (the annealing temperature was dependent on the primers), and (4) extension at 72 °C for 4 min (30 cycles in total). Next, 5 µL of the PCR product was verified by nucleic acid electrophoresis. After successful verification, the product was digested using 1 µL of DpnI with incubation at 37 °C for 1.5 h. The digested product was then transformed into the host *E. coli* BL21 cells, which were plated onto the kanamycin-resistant plates, and a single colony was randomly selected and cultured. The plasmids were extracted and sent to GENEWIZ (Wuxi, China) for sequencing. A correct sequencing result indicated the successful construction of the recombinant l-asparaginase mutant.

### 2.8. Characterization of the Enzymatic Properties of Recombinant l-asparaginase and Mutants

The optimal temperature was determined by measuring the enzyme activity at temperatures of 30, 35, 40, 45, 50, 55, and 60 °C for 10 min at pH 7.0. Each experiment was performed in triplicate.

The temperature stability was evaluated by incubating the purified enzyme in a water bath at 45 °C for 35 h. Samples were taken at various time points, and the residual enzyme activity was determined with respect to that at 0 h (defined as 100%).

The optimal pH was determined by measuring the enzyme activity at 45 °C for 10 min in various buffer solutions (acetate buffer for pH 4.0–6.0, Tris-HCl buffer for pH 7.0–8.0, and glycine-NaOH buffer for pH 9.0–10.0) containing a final l-asparagine concentration of 25 mmol·L^−1^. The highest enzyme activity was defined as 100%.

The pH stability was evaluated by incubating the purified enzyme in various buffer solutions (acetate buffer for pH 5.0–6.0, Tris-HCl buffer for pH 7.0–8.0, and glycine-NaOH buffer for pH 9.0–10.0) at 4 °C for 15 h. The residual enzyme activity was then measured at 45 °C and pH 7.0 at 0 h (defined as 100%).

The influence of metal ions on l-asparaginase activity was determined by measuring at 45 °C and pH 7.0 in the presence of various metal ions (Na^+^, Mg^2+^, La^3+^, Zn^2+^, Li^+^, Ca^2+^, K^+^, Mn^2+^, Ni^2+^, Co^2+^, Cu^2+^, or Fe^3+^) at a final concentration of 1 mmol·L^−1^. The Tris-HCl buffer system without any metal ions was used as a blank control.

### 2.9. Fermentation in a 5-L Fermenter

The recombinant *B. subtilis* strain was inoculated into 10 mL of LB liquid medium containing 50 μg/mL kanamycin and cultured at 37 °C and 200 rpm for 12 h. Then, 4 mL of this starter culture was transferred to 200 mL of LB medium containing 50 μg/mL of kanamycin. The resulting culture was incubated at 37 °C for another 12 h. Then, 10% (*v*/*v*) of the culture was inoculated into a 5 L fermenter (T&J Bio-engineering, Shanghai, China) with 2 L of fermentation medium, an incubation temperature of 37 °C, aeration 4.0 vvm, speed 600 rpm, pH 7.0, and automatic control of pH by coupling 50% ammonia and supplemented medium (glycerin 400g/L, yeast extract 50g/L, peptone 25g/L). We controlled the feeding rate at 50 mL·h^−1^. On the other hand, we maintained the dissolved oxygen (DO) at 30% throughout the fermentation process by coupling the speed and aeration. The whole fermentation process lasted for 36 h; samples were taken every four hours and analyzed for biomass.

## 3. Results

### 3.1. Cloning and Expression of l-asparaginase Genes from Different Sources

**Figure 1:**
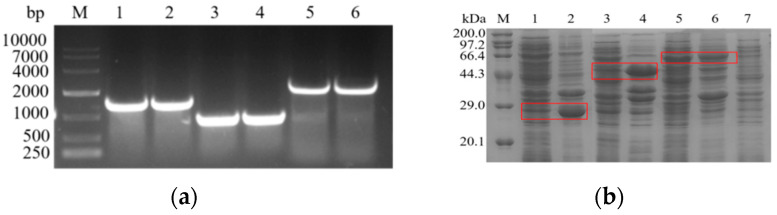
(**a**) Lane M: 10000 DNA marker; lanes 1 and 2: *Afans* gene; lanes 3 and 4: *Cjans* gene; lanes 5 and 6: *Rmans* gene; (**b**) SDS-PAGE analysis showing the results of l-asparaginase gene expression. Lane M: protein marker (kDa); lane 1: *E. coli* BL21/pET28a-*Cjans* crude enzyme; lane 2: *E. coli* BL21/pET28a-*Cjans* broken cell precipitate; lane 3: *E. coli* BL21/pET28a-*Afans* crude enzyme; lane 4: *E. coli* BL21/pET28a-*Afans* cell-breaking precipitate; lane 5: *E. coli* BL21/pET28a-*Rmans* crude enzyme; lane 6: *E. coli* BL21/pET28a-*Rmans* cell-breaking precipitate; lane 7: *E. coli* BL21/pET28a crude enzyme.

In the present study, we successfully cloned and amplified the l-asparaginase genes (*Afans*, *Cjans*, and *Rmans*) from *A. flavus* (1338bp), *C. jadinii* (771bp), and *R. miehei* (2043bp), respectively (Figure 1a). By ligating the cloned genes into the pET-28a plasmid and transforming them into *E. coli* BL21 for expression, we successfully constructed the recombinant l-asparaginase strains *E. coli* BL21/pET28a*-Afans*, *E. coli* BL21/pET28a-*Cjans*, and *E. coli* BL21/pET28a-*Rmans*. SDS-PAGE analysis (Figure 1b) indicated that the *Afans* (coding a 49.5kDa protein) and *Cjans* (coding a 28.5kDa protein) genes were expressed as inclusion bodies in *E. coli*, and the *Rmans* (coding a 75kDa protein) gene could be expressed normally in *E. coli*. The crude enzyme solutions were subjected to enzyme activity assays. The results revealed that the *E. coli* BL21/pET28a-*Afans* and *E. coli* BL21/pET28a-*Cjans* strains had no l-asparaginase activity, whereas the activity of *E. coli* BL21/pET28a-*Rmans* was approximately 4.1 U·mL^−1^.

### 3.2. Selection of Mutagenesis Sites for Improving Thermostability of l-asparaginase

**Figure 2:**
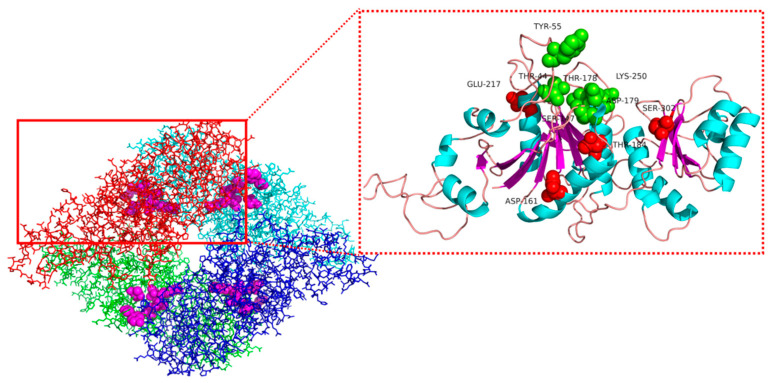
Three-dimensional structure of RmAsnase. The different colors indicate the four subunits of the RmAsnase tetramer, and the spheres highlight the enzyme active sites (green) and mutation sites (red) as expanded in the inset.

Protein structures are usually divided into four levels: polypeptide sequences are primary structures, helices and β-stands form secondary structures, 3D-folds are tertiary structures, and multiple polypeptide chains can be assembled into quaternary structures [26]. To guide our efforts to improve the activity and thermostability of l-asparaginase, we constructed a three-dimensional model structure based on the amino acid sequence of *R. miehei* l-asparaginase (RmAsnase) using the SWISS-MODEL server with the crystal structure of guinea pig l-asparaginase (PDB code: 4R8L) [27] as a template for homology modeling (Figure 2). Usually, modification of the flexible amino acid residues of the enzyme can improve thermal stability. Meanwhile, the flexible site within 5 Å of the catalytic triad has a significant effect on the catalytic activity [28]. Therefore, after avoiding the active center, we simulated the remaining flexible amino acid residues by FoldX. The protein unfolding free energy Δ*G* is a crucial thermodynamic parameter of proteins. The bioinformatics software FoldX was used to model the effects of amino acid mutations on Δ*G*. Specifically, the Δ*G* values were estimated before and after a particular RmAsnase mutation and used to calculate the corresponding unfolding free energy change, ΔΔ*G* = Δ*G*_mutant_ − Δ*G*_wild-type_ [28].

Suppose that the value of ΔΔ*G* for a particular mutation is less than zero. This would indicate that this mutation improves the thermostability of the enzyme, where more negative values correspond to greater stability [29]. Conversely, a ΔΔ*G* value greater than zero would indicate that this mutation is detrimental to enzyme stability. The mutants S302M, S302L, D161M, D184M, S302I, E217M, E217P, E217R, and S302V were constructed according to the order of ΔΔ*G* from smallest to largest (Table 1). Furthermore, the PYMOL software was used to examine the positions of the corresponding sites in the three-dimensional spatial structure of RmAsnase, avoiding the active center of the enzyme. In previous research by our group, we found that when the A344 site is mutated, the enzyme activity can be greatly improved [24]. Therefore, we introduced A344E as a mutation site to obtain a mutant with high activity.

### 3.3. Enzyme Activity Assays of Recombinant Mutants

We analyzed the enzyme activities of the recombinant l-asparaginase mutants. The specific enzyme activities of the mutants A344E, S302I, and S302M were increased by 55%, 32%, and 28%, respectively, compared with the wild-type enzyme (Table 2). We conducted further enzymatic property assays on these mutants exhibiting significantly increased activity.

### 3.4. Optimal Temperature and Thermostability of Recombinant Mutants

**Figure 3:**
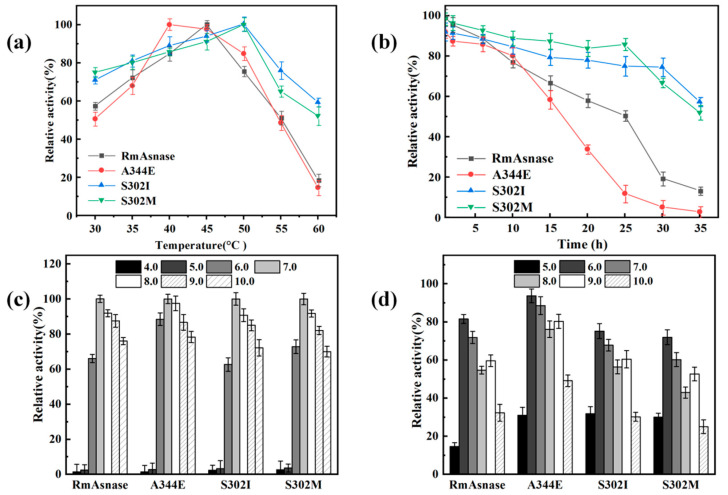
Effects of temperature and pH on the activity of wild-type *Rm*Asnase and its mutants: (**a**) optimal temperature, (**b**) thermostability at 45 °C, (**c**) optimal pH, and (**d**) pH stability.

The specific enzyme activities of the wild-type and mutant enzymes were measured at various temperatures (Figure 3a). We set the measured maximum enzyme activity of the wild-type enzyme and each mutant to 100% to investigate whether the optimal temperature changed. The optimal temperature of the recombinant wild-type enzyme was 45 °C, whereas the temperature of the A344E mutant had shifted to 40 °C. At 40–50 °C, the A344E mutant still exhibited high activity, retaining more than 80% of the initial activity. In contrast, the optimal temperature of the S302I and S302M mutants was 50 °C. At 50–60 °C, these mutants still retained more than 50% of their initial activities. 

We next examined the thermostability of the wild-type and mutant enzymes at 45 °C (Figure 3b). The residual activity of the wild-type enzyme after 20 h of heat treatment at 45 °C was 58%, whereas that for the A344E mutant was only 33%. Thus, the results indicated that the mutation of A344 affected the thermostability of l-asparaginase. After incubating the wild-type and A344E enzymes at 45 °C for 35 h, the residual enzyme activities were only 10% and 3%, respectively. In contrast, the S302I and S302M mutants retained over 50% of their initial activities.

### 3.5. Optimal pH and pH Stability of Recombinant Mutants

The pH is an important parameter influencing the activity and stability of enzymes. An appropriate pH promotes the enzymatic reaction, whereas an inappropriate pH can damage the enzyme structure and thus affect catalysis. As shown in Figure 3c, the highest activity was observed at pH 7.0 for both the wild-type and mutant enzymes. In contrast, the enzyme activity was negligible for pH values of 4.0–5.0. The relative activity of the A344E mutant was comparatively high, at pH 6.0–9.0, with respect to the recombinant enzyme, maintaining more than 80% of enzyme activity. The pH stability of the wild-type and mutant enzymes was also examined, as shown in Figure 3d. Similar trends were observed in all cases, although the A344E mutant displayed greater resistance to acids and bases compared with the wild-type and other mutant enzymes.

### 3.6. Influence of Metal Ions on l-asparaginase Activity

**Figure 4:**
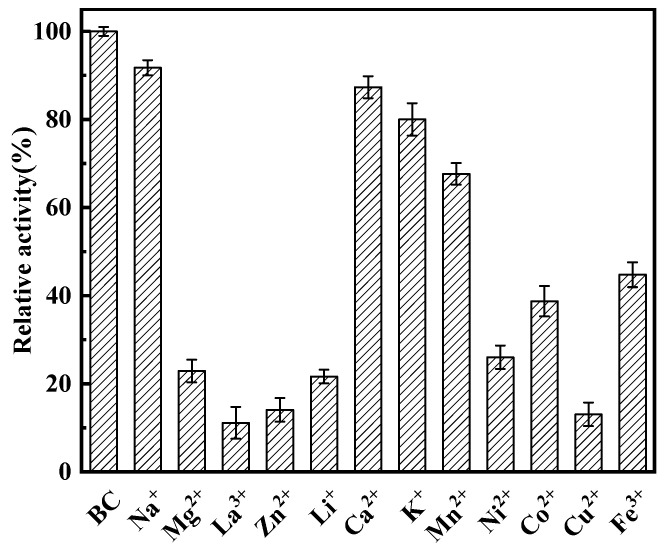
Effects of various metal ions on the activity of l-asparaginase (BC: blank control).

The effects of various metal ions (Na^+^, Mg^2+^, La^3+^, Zn^2+^, Li^+^, Ca^2+^, K^+^, Mn^2+^, Ni^2+^, Co^2+^, Cu^2+^, and Fe^3+^) on the catalytic activity of RmAsnase were investigated using 25 mmol·L^−1^ of l-asparagine as the substrate. We found that Cu^2+^, La^3+^, and Zn^2+^ exerted a significant inhibitory effect on l-asparaginase. In contrast, Na^+^ and Ca^2+^ had minimal effect on the enzyme, and none of the tested metal ions displayed an activating effect (Figure 4).

### 3.7. Structural Simulation and Analysis of l-asparaginase Mutants

**Figure 5:**
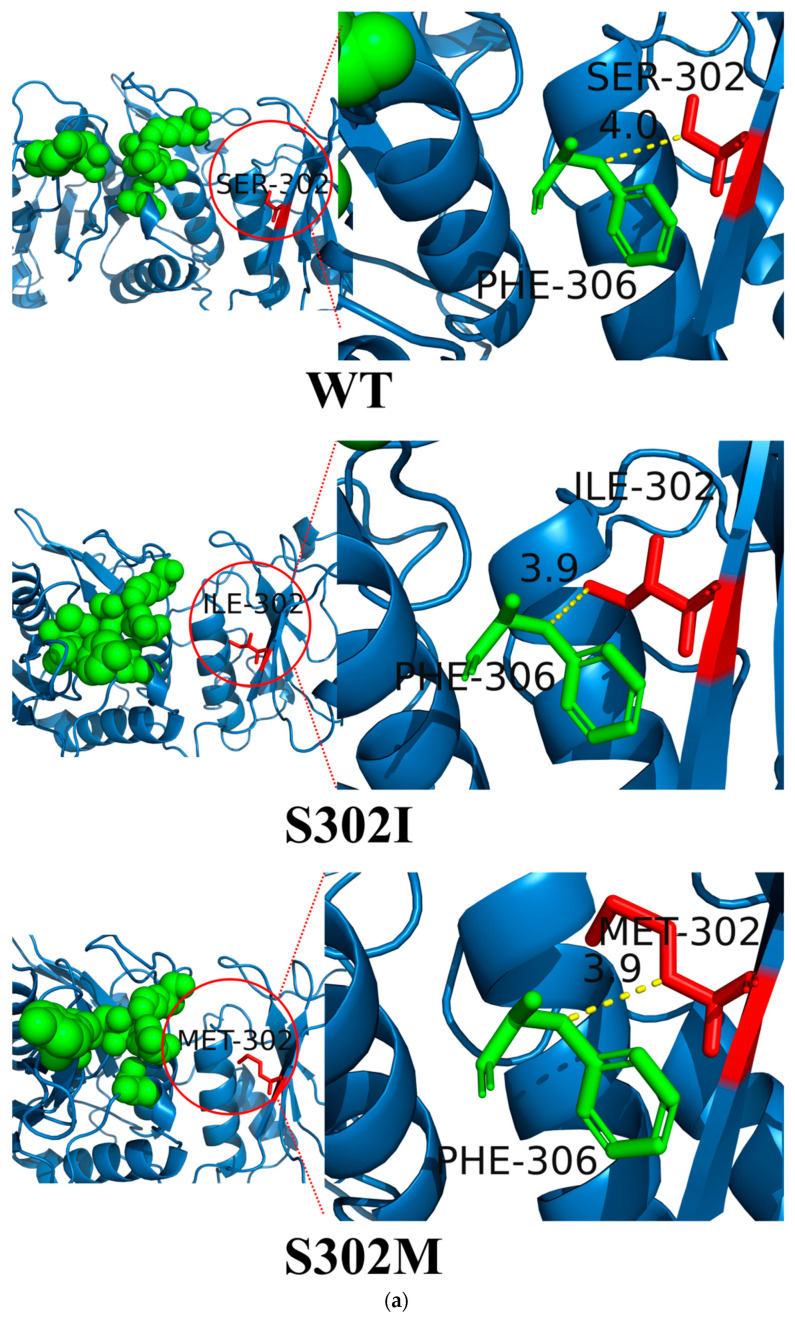

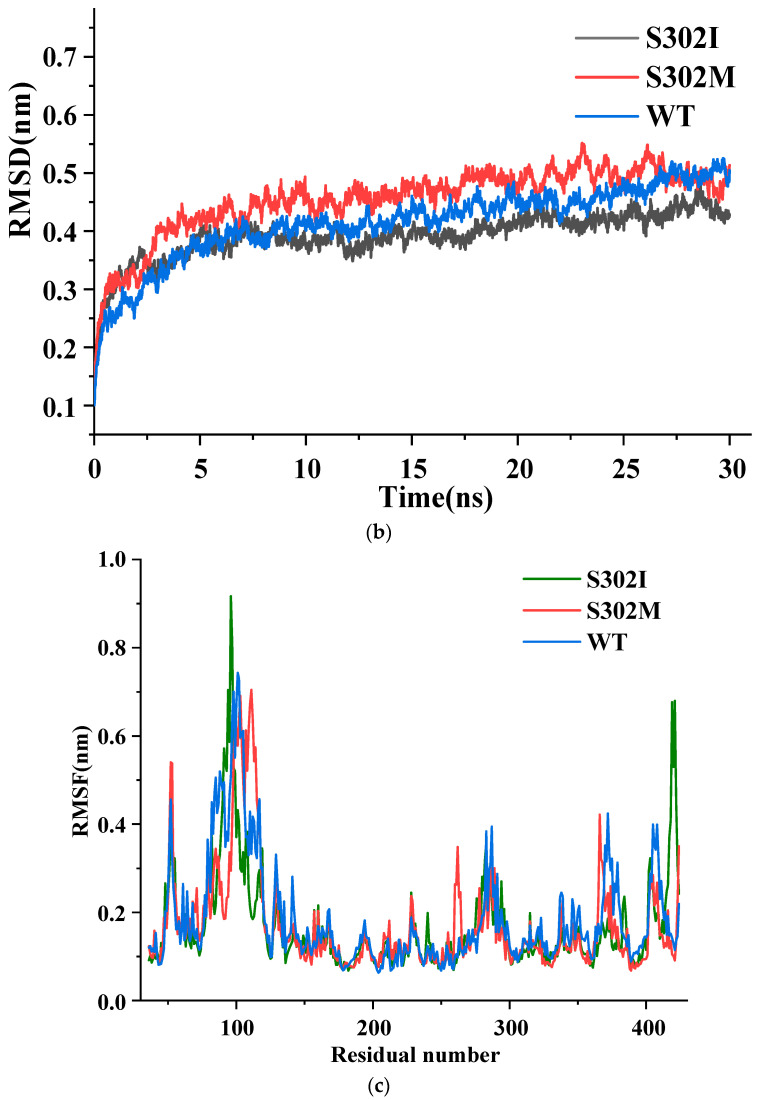
(**a**) Comparison of protein structures (the green sphere represents the active site of the enzyme, and the short yellow line represents the distance of two amino acid residues) for the (WT) wild-type enzyme, (S302I) S302I mutant, and (S302M) S302M mutant; (**b**) RMSD values; and (**c**) RMSF values.

To investigate the origin of the changes in thermostability observed for the mutant enzymes, we performed molecular dynamics simulations to examine the three-dimensional structural changes of RmAsnase before and after mutation. The results showed that S302 was located at the β-fold, and both I302 and M302 could still form the β-fold after mutation, which did not lead to significant changes in the protein structure (Figure 5a). The residues at position 302 did not form hydrogen bonds with the surrounding amino acids, and the number of hydrogen bonds remained unaltered after mutation. Several of the amino acid residues immediately adjacent to position 302, namely, V299, A301, and L303, were all hydrophobic amino acids. Thus, the mutation of hydrophilic serine to hydrophobic isoleucine or methionine may increase the hydrophobicity of this region and improve the protein stability. 

Meanwhile, we found that the amino acid residue at position 302 only interacts with the residue at position 307; after the mutation, the distance between these two amino acid residues in the mutant was shortened (Figure 5a). We can also see that S302 is adjacent to the enzyme active center (Figure 5a), and the side chains of both isoleucine and methionine are longer than that of serine. Thus, we speculate that the longer side chains in S302I and S302M resulted in less space to accommodate the substrate l-asparagine, which is conducive to proper enzyme–substrate binding and may account for the enhanced specific activity. We analyzed the dynamic behavior of the wild-type and mutants systems by 30 ns MD simulation. The stability of the systems is characterized by the RMSD value. As shown in Figure 5b, these three systems have the same fluctuating trend. The systems fluctuate between 0 and 0.5 nm until equilibrium is reached at 6 ns. This indicates that the simulated trajectories of 8 to 30 ns are stable and representative. 

Thus, to further investigate the reason for the improved thermostability, the protein molecular dynamics were simulated using the GROMACS software to determine the root-mean-square fluctuation (RMSF), which is an indicator of protein stability. The results are presented in Figure 5c. Some regions around residue S302 show large fluctuations in the RMSF values of WT at 318 K. Usually, these amino acid residues are considered unstable. In contrast, the RMSF values of the two mutants exhibited more minor fluctuations. On the other hand, the average RMSF values of mutants S302I (0.167) and S302M (0.170) were lower than those of WT (0.184). The decreased RMSF values explain why the mutants S302M and S302I are more thermostable.

### 3.8. Construction of Combination Mutants and Determination of General Properties

To obtain enzyme mutants with superior activity and thermostability, we combined the above positive mutants A344E, S302I, and S302M to construct the combination mutants A344E/S302I and A344E/S302M. The resulting activities are listed in Table 3. The specific activities of the combination mutants A344E/S302I and A344E/S302M increased by 43.8% and 39.2%, respectively, compared with the wild-type enzyme.

**Figure 6:**
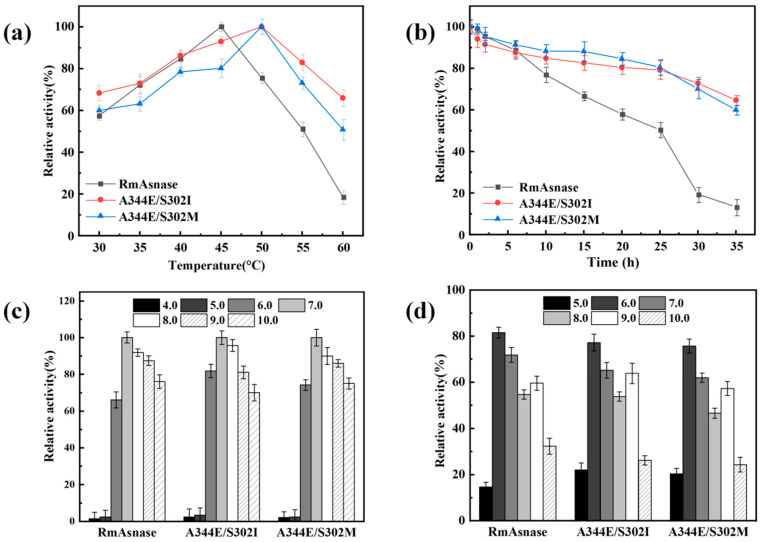
Effects of temperature and pH on the activity of the combination mutants: (**a**) optimal temperature; (**b**) thermostability at 45 °C; (**c**) optimal pH; and (**d**) pH stability.

Further investigation of the enzymatic properties of the combination mutants revealed that the optimal temperature of the A344E/S302I and A344E/S302M mutants was 50 °C, which is 5 °C higher than that of the wild-type enzyme (Figure 6a). The relative enzyme activities of A344E/S302I and A344E/S302M were higher than those of the wild-type enzyme in the range of 50–60 °C. Furthermore, A344E/S302I and A344E/S302M were more thermostable than the wild-type enzyme at 45 °C (Figure 6b). After incubation at 45 °C for 35 h, the residual enzyme activities of A344E/S302I and A344E/S302M were approximately 65% and 60%, respectively. In contrast, the wild-type enzyme retained only about 10% of its initial activity.

The optimal pH was 7.0 for both the combination mutants and the wild-type enzyme (Figure 6c). Moreover, similar trends were observed over the tested pH range of 4.0–10.0 in all cases. In pH stability experiments, all three enzymes also displayed similar results (Figure 6d).

### 3.9. Construction of a High-Yield l-asparaginase B. subtilis Recombinant and Production in a 5-L Fermenter

*B. subtilis* is a common model system that is relatively well understood and generally considered safe for use in food production, as it does not produce toxic byproducts during fermentation [30]. As such, we selected *B. subtilis* as a host for l-asparaginase production. Based on the above results, the recombinant plasmid pMA5-A344E/S302I was constructed and transformed into *B. subtilis* 168 to obtain *B. subtilis* 168-pMA5-A344E/S302I.

However, the enzyme activity of *B. subtilis* 168-pMA5-A344E/S302I was at a low level. Xiao et al. [31] designed a 5′ untranslated region (UTR) sequence that effectively increased the protein yield of the industrial strain *B. licheniformis* DW2. This sequence contains only about 30 nt and forms a hairpin structure before the open reading frame. This element was reported to increase the expression of target proteins such as nattonase and keratinase and to facilitate the accessibility of Shine–Dalgarno sequences and start codons, thus improving the translation initiation efficiency. Therefore, we attempted to apply the UTR strategy to enhance the expression of l-asparaginase in *B. subtilis.* The assay results revealed that the enzyme activity of *B. subtilis* 168-pMA5-UTR-A344E/S302I was significantly increased from 2.1 U·mL^−1^ in the wild-type strain to 13.3 U·mL^−1^. Thus, the UTR modification strategy effectively increased the l-asparaginase expression in *B. subtilis.*

**Figure 7:**
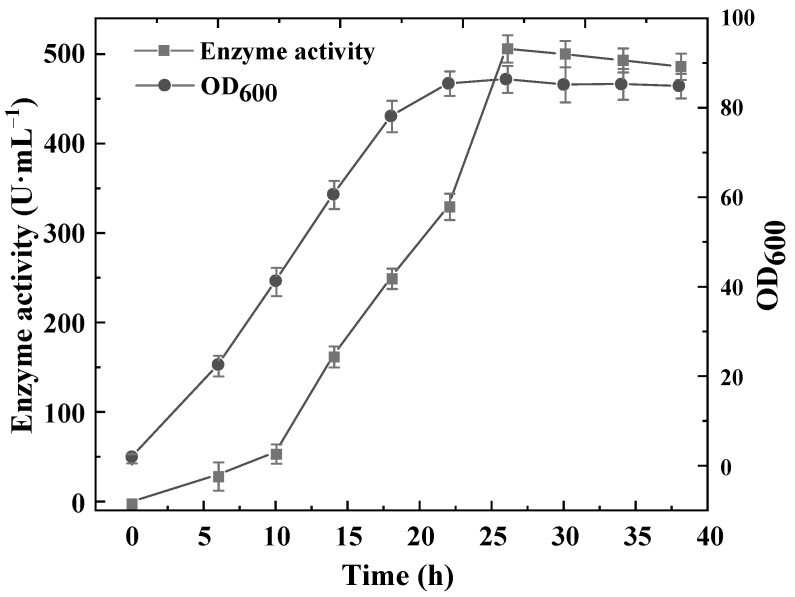
Fermentation results for *B. subtilis* 168-pMA5-UTR-A344E/S302I in a 5-L fermenter.

We further fermented the recombinant *B. subtilis* 168-pMA5-UTR-A344E/S302I strain in a 5-L bioreactor. The fermentation results are presented in Figure 7, where it is shown that the recombinant strain reached the stable phase during 22–26 h of fermentation. After 30 h, the recombinant bacteria entered the decay phase. For each stage of fermentation, the production of l-asparaginase increased with increasing bacterial biomass, and when the bacterial biomass reached its maximum after 26 h of fermentation the l-asparaginase activity was as high as 521.9 U·mL^−1^.

## 4. Discussion

Enzyme preparations have been extensively applied in the food, chemical, and pharmaceutical industries. However, these applications have been hindered by the complex environments typically encountered in these industries, owing to the poor thermostability of enzymes. Rational design methods have shown advantages in prediction and the modification of enzymes structures to improve their thermostability. Currently, the combination of protein engineering and rational design methods based on computer simulation of protein models has been applied to many industrial enzymes, which completes protein engineering and makes this method more precise and efficient. For example, Zhang et al. [32] used POPMuSiC to calculate the free energy change and obtained a lipase mutant with a 9.6-fold increase in half-life. Yang et al. [33] enhanced the thermostability of alginate lyase cAlyM by using a rational design method; the mutants showed an increase by 2.25 h in half-life time at 45 °C. Liu et al. [34] improved thermostability via silico rational design and systems engineering; the half-life of the alkaline alpha-amylases at high temperature was increased by 6-fold, while its optimum temperature was increased by 5 °C.

l-asparaginase has a wide range of applications in food industries; however, the complexity of the working environment in the pharmaceutical industry requires the enzyme to maintain high catalytic activity over a wide pH and temperature range. The optimum reaction conditions of the reported l-asparaginase do not match the commercial application conditions in terms of temperature and pH preference. Li et al. [23] performed heterologous expression of l-asparaginase from different sources. The specific enzyme activity of l-asparaginase from thermophilic bacteria was high. However, there was no enzyme activity below 60 °C, which was challenging in meeting industrial applications (good thermal stability of l-asparaginase at 40–50 °C).

Based on previous studies, we focused on l-asparaginase with temperatures in the range of 40–50 °C and pH of 6.0–9.0. We heterologously expressed the l-asparaginase gene from *Rhizomucor miehei* and constructed a recombinant strain *E. coli* BL21/pET28a-*Rmans* in this study. However, we found that RmAsnase was less thermostable at 40 °C and 45 °C. To address the disadvantages of poor thermostability at 45 °C, we found that site-directed mutagenesis can effectively improve the enzymatic properties; for example, Verma et al. [35] subjected surface amino acid residues of *E. coli* l-asparaginase to fixed point mutation and obtained several mutants with enhanced thermostability. They found that EcA(Y176F) leads to a considerably higher value of ΔG(H_2_O) as compared to EcA(WT). Long et al. [36] constructed the G107D mutant of a *B. subtilis* l-asparaginase by fixed-point mutation, which exhibited enhanced catalytic activity and thermostability. Sudhir et al. [37] examined the role of residues outside of the active center for *Bacillus licheniformis* l-asparaginase and observed that the D103V mutant displayed a threefold increase in half-life and heat resistance, in addition to high substrate affinity. 

Therefore, we rationally designed and modified *Rm*Asnase based on the FoldX algorithm and PYMOL software analysis to obtain a recombinant strain of l-asparaginase with high enzyme activity and thermostability; the optimum temperature of combination mutants A344E/S302I was 5 °C higher compared with the wild-type enzyme, and more than 60% of the enzyme activity was retained after 35 h at 45 °C. The specific enzyme activity was at a higher level when compared with most non-thermophilic sources of l-asparaginase. After determining the influence of metal ions on recombinant asparaginase, we found that Na^+^, Ca^2+^ had only slight inhibitory effects on l-asparaginase activity; however, La^3+^, Cu^2+^, Zn^3+^ strongly inhibited l-asparaginase activity, which is consistent with the findings of Huang et al. [38].

Then, a high-yield l-asparaginase strain *B. subtilis* 168-pMA5-A344E/S302I was constructed, and the crude enzyme activity of l-asparaginase was increased from 2.1 U·mL^−1^ to 13.3 U·mL^−1^ by 5′ untranslated region (UTR) modification. In a 5-L fermenter scale-up experiment, the enzyme activity of recombinant *B. subtilis* 168-pMA5 UTR-A344E/S302I was 39-fold higher under high density culture.

## 5. Conclusions

In this study, we mutated the amino acid residue 302 of l-asparaginase derived from *Rhizomucor miehei* by rational design. We obtained mutants S302I and S302M by mutating hydrophilic serine to hydrophobic isoleucine and methionine. After measuring the enzymatic properties of the mutants, we found that the thermal stability of mutants S302I and S302M was significantly enhanced compared to the original RmAsnase. The thermal stability of mutants A344E/S302I and A344E/S302M was further enhanced after the combination mutation. We selected *B. subtilis* 168 as the expression host and greatly improved the expression of RmAsnase by 5′ untranslated region modification. In the final 5-L fermenter scale-up study, the recombinant strain achieved an enzyme activity of 521.9 U·mL^−1^ after 36 h of fed-batch fermentation.

## Figures and Tables

**Table 1:** The ΔΔ*G* values of candidate mutants computed by FoldX.

Position	Original Amino Acid	Mutant Amino Acid	ΔΔ*G* Value (kcal·mol^−1^)
302	S	M	−2.42261
302	S	L	−2.39485
161	D	M	−1.94591
184	D	M	−1.69645
302	S	I	−1.61742
217	E	M	−1.57523
217	E	P	−1.56941
217	E	R	−1.41972
302	S	V	−1.17737

**Table 2:** Comparison of enzyme activity for the wild-type and mutant enzymes.

Enzyme	Specific Enzyme Activity (U·mg^−1^)	Relative Enzyme Activity (100%)
WT	509.1 ± 0.5	100.0 ± 1.0
A344E	786.8 ± 0.7	154.5 ± 1.4
S302I	672.2 ± 0.3	132.0 ± 0.6
S302M	650.1 ± 0.9	127.7 ± 1.8
E217R	581.5 ± 0.3	114.2 ± 0.7
E217P	571.4 ± 0.5	112.2 ± 1.1
S302V	412.6 ± 0.6	81.0 ± 1.3
S302L	410.9 ± 0.7	80.7 ± 1.5
E217M	152.1 ± 1.0	29.9 ± 2.1
D161M	100.3 ± 0.6	19.7 ± 1.3
D184M	82.3 ± 0.9	16.2 ± 1.8

**Table 3:** Comparison of enzyme activity for the wild-type and combination mutant enzymes.

Enzyme	Specific Enzyme Activity (U·mg^−1^)	Relative Enzyme Activity (100%)
WT	509.1 ± 0.6	100.0 ± 1.2
A344E/S302I	732.2 ± 0.3	143.8 ± 0.7
A344E/S302M	708.9 ± 1.2	139.2 ± 2.5

## Data Availability

The data that support the findings of this study are available from the corresponding author upon reasonable request.

## Supplementary Materials

The following are available online at https://www.mdpi.com/article/10.3390/biology10121346/s1: Table S1, strains and plasmids used in this study; Table S2, main primers used in this study; Table S3, Optimum temperature; Table S4, Thermal stability at 45 °C; Table S5, Optimum pH; Table S6, pH stability; Figure S1, The DO and pH curves.
