# Supplementary material for: Heterologous Expression and Rational Design of l-asparaginase from Rhizomucor miehei to Improve Thermostability
Source: Biology (Basel). 2021 Dec 17;10(12):1346. doi: 10.3390/biology10121346 (PMC8698271; doi:10.3390/biology10121346)

Table S1: strains and plasmids used in this study

strain and plasmid	description	source or reference
E. coli BL21(DE3)	expression host	laboratory [illegible]
E. coli BL21/pET28a-*Rmasn*	*E. coli* BL21 derivative with pET28a-*Rmasn*,Kan^{r}	this work
E. coli BL21/pET28a-*Afasn*	*E. coli* BL21 derivative withpET28a-*Afasn*,Kan^{r}	this work
E. coli BL21/pET28a-*Cjasn*	*E. coli* BL21 derivative withpET28a-*Cjasn*,Kan^{r}	this work
E. coli BL21/pET28a-A344E	*E. coli* BL21 derivative with ET28a-A344E,Kan^{r}	this work
E. coli BL21/pET28a-S302M	*E. coli* BL21 derivative with pET28a- S302M,Kan^{r}	this work
E. coli BL21/pET28a-S302L	*E. coli* BL21 derivative with pET28a- S302L,Kan^{r}	this work
E. coli BL21/pET28a-D161M	*E. coli* BL21 derivative with pET28a- D161M,Kan^{r}	this work
E. coli BL21/pET28a-D184M	*E. coli* BL21 derivative with pET28a-D184M,Kan^{r}	this work
E. coli BL21/pET28a-S302I	*E. coli* BL21 derivative with pET28a-S302I,Kan^{r}	this work
E. coli BL21/pET28a-E217M	*E. coli* BL21 derivative with pET28a-E217M,Kan^{r}	this work
E. coli BL21/pET28a-E217P	*E. coli* BL21 derivative with pET28a-E217P,Kan^{r}	this work
E. coli BL21/pET28a-E217R	*E. coli* BL21 derivative with pET28a-E217R,Kan^{r}	this work
E. coli BL21/pET28a-S302V	*E. coli* BL21 derivative with pET28a-S302V,Kan^{r}	this work
E. coli BL21/pET28a-A344E/S302I	*E. coli* BL21 derivative with pET-8a-A344E/S302I,Kan^{r}	this work
E. coli BL21/pET28a-A344E/S302M	*E. coli* BL21 derivative with pET28a-A344E/S302M,Kan^{r}	this work
E. coli JM109	cloned host	laboratory [illegible]
B. subtilis 168	cloned host	laboratory stock

Table S1:bacterial strains and plasmids used in this study

strain and plasmid	description	source or reference
B. subtilis 168/pMA5-A344E/S302I	*B. subtilis* 168 derivative with pMA5-A344E/S302I,Kan^r	this work
B. subtilis 168/pMA5 UTR-A344E/S302I	*B. subtilis* 168 derivative with pMA5 UTR-A344E/S302I,Kan^r	this work
pET-28a-*Rmasn*	pET-28a derivative with *Rmasn*,Kan^r	this work
pMA5-A344E/S302I	pMA5 derivative with A344E/S302I, Amp^r (*E. coli* BL21) / Kan^r (*B. subtilis* 168)	this work
pMA5 UTR-A344E/S302I	pMA5 derivative with UTR-E/S302I,Amp^r (*E. coli* BL21) / Kan^r (*B. subtilis* 168)	this work

Table S2 Main primers used in this study

Primer	sequence (5′→3′)
AFA-F	GTGCCGCGCGGCAGCCATATGATGGCTGAACCAAAACCTAA (*Nde* I)
AFA-R	GTGGTGGTGGTGGTGCTCGAGTTAGTATGGTTCACGGAAAA (*Xho* I)
CJA-F	GTGCCGCGCGGCAGCCATATGATGACTATCTCTCACCCAGA (*Nde* I)
CJA-R	GTGGTGGTGGTGGTGCTCGAGTTATTGTCCACCACCGTAGA (*Xho* I)
RMA-F	TGGTGCCGCGCGGCAGCCATATGGATTCTCGTACTACAGCTCA (*Nde* I)
RMA-R	TGGTGGTGGTGGTGGTGCTCGAGTTCTTTACCAAGAAGTTGAG (*Xho* I)
A344E-F	CTTTTAGCTGAATTAAAAGAAGCTTGTGATCGTGGAGTTGTA

A344E-R	TCTTTTAATTCAGCTAAAAGACCTTGACGAGCAGGTGC
S302I-F	ACGTAGCTATCTTACGTTTATTCCCAGGCATTAACGAGAGC
S302I-R	TAAACGTAAGATAGCTACGTTACGGTTAAGAACTTTGTGAGAACG
S302M-F	AACGTAGCTATGTTACGTTTATTCCCAGGCATTAACGAGAGCA
S302M-R	AAACGTAACATAGCTACGTTACGGTTAAGAACTTTGTGAGAACGAA
pMA5-RMA-F	TGCAAAAAGTGAAATCAGGGGGATCCATGGATTCTCGTACTACAGCTCACGTACC (*Bam*H I)
pMA5-RMA-R	GTGAATTTCGACCTCTAGAACGCGTTTATTCTTTACCAAGAAGTTGAGCGATTTC (*Mlu* I)

Table S3 Optimum temperature

Temperature(℃)	Wild-type(U·mg⁻¹)	A344E(U·mg⁻¹)	S302M(U·mg⁻¹)	S302I(U·mg⁻¹)	A344E/S302M (U·mg⁻¹)	A344ES302I(U·mg⁻¹)
30	292.5±1.2	398.7±1.4	477.3±0.8	487.6±2.3	500.2±0.7	425.1±0.9
35	367.6±0.5	535.5±0.9	544.6±0.6	520.7±0.6	534.5±0.6	448.0±0.8
40	431.2± 0.7	787.2±0.8	598.5±0.8	557.4±1.4	632.0±0.8	556.0±0.6
45	509.2± 1.3	768.4±0.8	631.9±0.2	592.2±1.3	680.9±0.6	568.5±2.1
50	384.6± 0.9	667.8±0.9	674.6±0.6	650.1±0.6	732.9±0.9	709.6±1.6
55	260.7±1.5	383.1±1.3	510.9±0.7	423.2±0.7	607.7±1.6	518.8±1.4
60	95.3± 1.6	115.6±1.5	399.0±0.9	339.8±1.2	483.1±0.6	360.4±0.6

Table S4 Thermal stability at 45℃

Time (h)	Wild-type(U·mg^{-1})	A344E(U·mg^{-1})	S302M(U·mg^{-1})	S302I(U·mg^{-1})	A344E/S302M(U·mg^{-1})	A344ES302I(U·mg^{-1})
1	503.7±1.6	724.5±0.6	639.1±1.3	618.2±0.6	689.3±0.9	703.9±1.8
2	485.8±2.3	687.6±2.5	626.4±2.4	614.1±1.3	669.9±1.4	675.9±2.3
6	451.6±2.6	675.1±1.4	602.6±1.6	594.7±2.1	641.6±0.8	647.9±0.6
10	391.6±1.8	631.0±1.6	577.1±1.2	568.8±1.6	621.0±0.7	626.1±2.3
15	339.5±1.2	459.8±2.1	567.9±2.3	533.0±1.4	605.0±2.1	624.8 ±0.9
20	295.1±0.9	266.7±1.9	544.7±0.9	524.2±1.9	588.9±1.4	599.9±1.3
25	256.8±0.7	93.8±1.6	557.4±2.5	504.2±1.8	580.0±2.6	570.7±1.2
30	98.7±1.5	40.7±2.3	434.1±1.3	500.7±1.3	533.0±3.1	496.6±2.1
35	67.9±2.1	22.1±0.7	338.1±1.6	385.5±1.6	473.7± 2.4	425.4±0.9

Table S5 Optimum pH

pH	Wild-type (U·mg^{-1})	A344E(U·mg^{-1})	S302M(U·mg^{-1})	S302I(U·mg^{-1})	A344E/S302M(U·mg^{-1})	A344ES302I(U·mg^{-1})
4	7.0±1.3	11.4±0.6	17.3±0.8	15.7±2.3	17.4±1.7	15.4±2.6
5	12.5±2.1	21.5±1.5	23.1±1.4	21.7±2.6	25.3±1.5	17.4±1.6
6	336.6±0.9	695.9±2.1	473.2±1.3	420.9±1.4	598.9±0.9	525.9±2.8
7	509.7±1.7	786.9±1.7	650.1±1.9	672.3±0.9	732.6±2.7	709.1±1.3
8	467.9±2.5	767.2±0.9	596.4±2.9	610.4±1.6	701.0±0.8	637.9±2.4
9	445.4±1.2	681.9±0.6	533.5±2.4	571.4±1.7	593.6±1.5	609.4±0.9
10	387.1±0.6	616.5±0.5	455.1±2.3	484.7±0.6	512.5±2.3	531.7±1.6

Table S6 pH stability

pH	Wild-type (U·mg⁻¹)	A344E(U·mg⁻¹)	S302M(U·mg⁻¹)	S302I(U·mg⁻¹)	A344E/S302M(U·mg⁻¹)	A344ES302I(U·mg⁻¹)
5	74.4±1.6	243.8±2.6	213.9±0.9	194.9±0.9	161.4±1.6	144.5±0.9
6	415.2±0.7	737.0±1.3	505.2±2.5	467.7±1.5	565.2±2.0	536.4±1.9
7	365.5±1.9	696.9± 2.4	455.6± 3.1	391.4±2.4	476.8±3.6	438.9±2.5
8	278.3±2.3	598.8±1.6	379.1± 0.9	278.9±1.7	393.5±0.7	330.4±1.4
9	303.3±1.4	631.5±0.7	405.9±2.5	342.2±2.1	467.2±1.2	405.8±3.0
10	164.3±1.8	386.3±0.7	202.7±1.3	162.4±0.9	191.4±1.0	172.2±2.6

Figure S1 The DO and pH curves

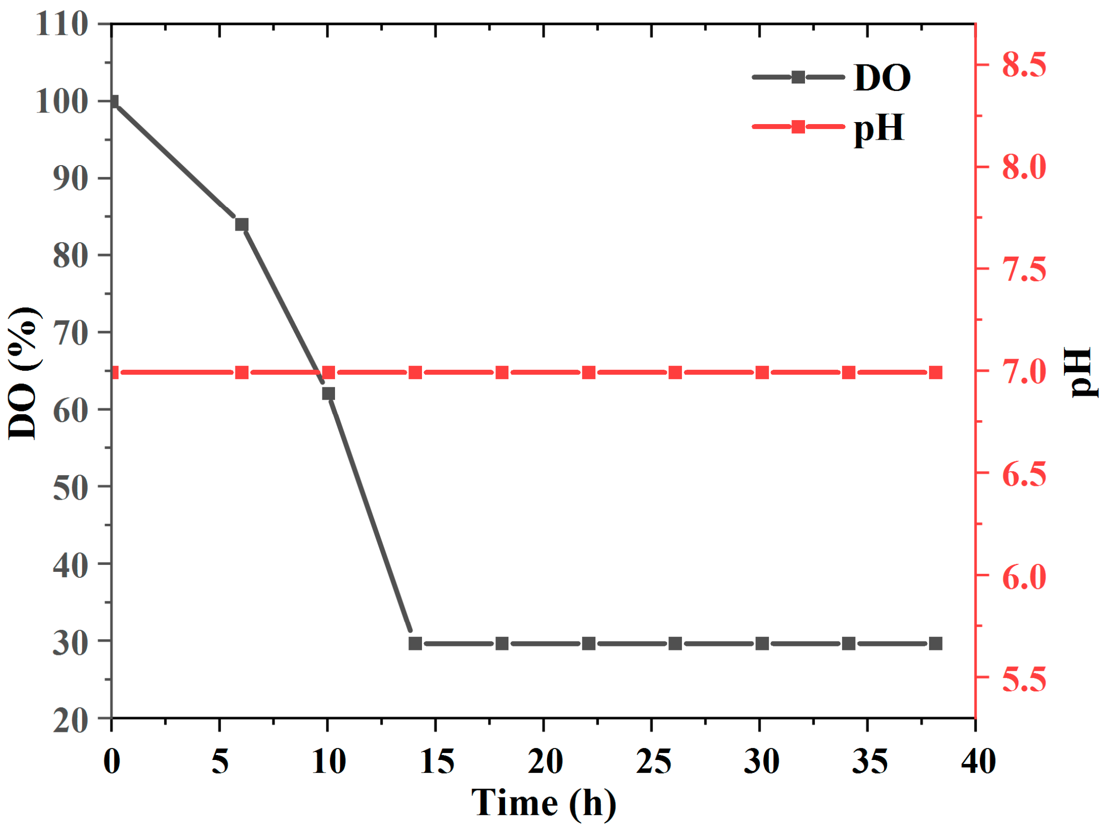

Supplement: Supplementary file 1 [file biology-10-01346-s001.zip › biology-1499778-supplementary.pdf]
